# Stimulus-responsive block copolymer nano-objects and hydrogels *via* dynamic covalent chemistry†
†Electronic supplementary information (ESI) available: GPC date, additional TEM images and DLS data. See DOI: 10.1039/c7py01242j



**DOI:** 10.1039/c7py01242j

**Published:** 2017-07-28

**Authors:** Renhua Deng, Yin Ning, Elizabeth R. Jones, Victoria J. Cunningham, Nicholas J. W. Penfold, Steven P. Armes

**Affiliations:** a Dainton Building , Department of Chemistry , The University of Sheffield , Brook Hill , Sheffield , South Yorkshire S3 7HF , UK . Email: rhd.deng@gmail.com ; Email: s.p.armes@sheffield.ac.uk

## Abstract

Herein we demonstrate that dynamic covalent chemistry can be used to induce reversible morphological transitions in block copolymer nano-objects and hydrogels. Poly(glycerol monomethacrylate)–poly(2-hydroxypropyl methacrylate) (PGMA–PHPMA) diblock copolymer nano-objects (vesicles or worms) were prepared *via* polymerization-induced self-assembly. Addition of 4-carboxyphenylboronic acid (CPBA) leads to the formation of phenylboronate ester bonds with the 1,2-diol pendent groups on the hydrophilic PGMA stabilizer chains; such binding causes a subtle reduction in the packing parameter, which in turn induces either vesicle-to-worm or worm-to-sphere transitions. Moreover, CPBA binding is pH-dependent, so *reversible* transitions can be achieved by switching the solution pH, with relatively high copolymer concentrations leading to associated (de)gelation. This distinguishes these new physical hydrogels from the covalently cross-linked gels prepared using dynamic covalent chemistry reported in the literature.

## Abstract 2

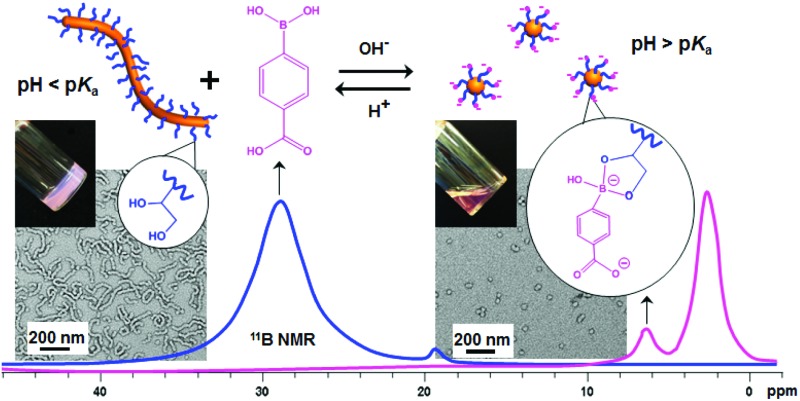
Dynamic covalent chemistry can be used to induce reversible morphological transitions for block copolymer nano-objects in mildly alkaline solution.

## Introduction

Stimuli-responsive block copolymer nano-objects are attractive owing to their potential as smart materials for various applications.^1–4^ An important design principle for many examples of stimulus-responsive vesicles, worms or spheres reported in the literature involves the tunable hydrophobic–hydrophilic nature of the membrane- or core-forming block.^5–9^ Recently, a considerable body of work has focused on examining morphological transformations such as vesicle-to-sphere and worm-to-sphere transitions *etc*.^10–14^ The former can be used for the *in situ* release of payloads,^15–19^ while the latter enables the design of smart physical hydrogels.^20^ Typically, the desired change in copolymer morphology involves a subtle change in the fractional packing parameter^21^ arising from variation in the relative volume fractions of the steric stabilizer and structure-directing blocks. Most of the morphological transformations described in the literature are triggered by changes in either temperature^22–28^ and/or pH.^20,29–32^ However, in only a few cases is such a response elicited *via* molecular recognition, whereby the analyte of interest induces a morphological transition by selective binding with the steric stabilizer chains.^33,34^ On the other hand, dynamic covalent chemistry has been widely exploited for the design of stimulus-responsive polymers.^35–38^ In this context, boronic acid-based (co)polymers have recently become the subject of significant attention owing to their ability to form dynamic boronate ester covalent bonds with either 1,2- or 1,3-diols.^39–51^ For example, the molecular dissociation of boronic acid-based copolymer micelles can be triggered *via* binding to small molecules such as glucose, which offers a potential therapy for diabetes.^49–51^ Conversely, we recently reported that vesicle-to-worm transitions of block copolymer containing 1,2-diol groups can be triggered by binding to a water-soluble phenylboronic acid derivative (3-aminophenylboronic acid).^52^ In addition to selectivity, one key feature of such dynamic covalent chemistry is its reversibility. However, as far as we are aware, using boronate ester covalent bonds to induce *reversible* morphological transitions in block copolymer nano-objects has not yet been reported.

**Scheme 1:**
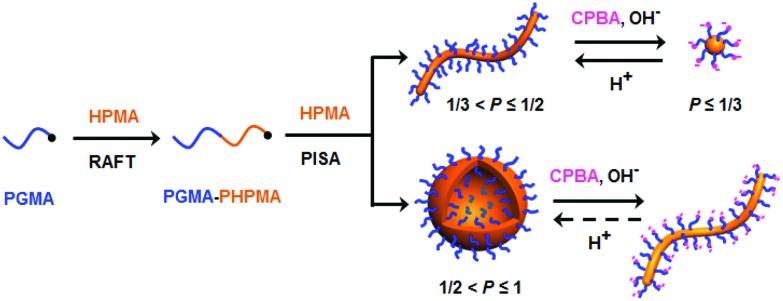
Synthesis of PGMA_45_–PHPMA_*x*_ diblock copolymer nano-objects *via* RAFT aqueous dispersion polymerization (where *P* is the fractional packing parameter^21^) and their subsequent morphological transitions driven by switching pH in the presence of CPBA.

In the present work, we demonstrate that dynamic covalent chemistry can be utilized to induce reversible morphological transformations in block copolymer nano-objects (see Scheme 1). More specifically, poly(glycerol monomethacrylate)–poly(2-hydroxypropyl methacrylate) (PGMA–PHPMA) vesicles or worms were prepared in the form of concentrated aqueous dispersions *via* polymerization-induced self-assembly (PISA).^53–55^ On addition of 4-carboxyphenylboronic acid (CPBA), reversible vesicle-to-worm or worm-to-sphere transitions were observed in mildly alkaline solution on switching pH. Moreover, reversible (de)gelation occurs if these experiments are conducted at 10% w/w copolymer, rather than in dilute solution. Such physical hydrogels differ markedly from various previously reported cross-linked gels formed using dynamic boronate ester chemistry.^40,46,48^

## Experimental

### Materials

Glycerol monomethacrylate (GMA; 99.8%) was donated by GEO Specialty Chemicals (Hythe, UK) and was used without further purification. 2-Hydroxypropyl methacrylate (HPMA) was purchased from Alfa Aesar (UK) and was used as received. 4,4′-Azobis(4-cyanopentanoic acid) (ACVA; V-501; 99%), 2,2′-azobisisobutyronitrile (AIBN), 2-cyano-2-propyl dithiobenzoate (CPDB), 4-carboxyphenylboronic acid (CPBA; ≥90%), ethanol (99%, anhydrous grade), methanol, and dichloromethane were purchased from Sigma-Aldrich (UK) and were used as received. Deuterated methanol (CD_3_OD) was purchased from Goss Scientific (Nantwich, UK). Buffer solutions were purchased from Thermo Fisher Scientific (Chelmsford, USA). All solvents were HPLC-grade and were purchased from Fisher Scientific (Loughborough, UK).

### Synthetic procedures

#### Synthesis of PGMA_45_ macro-CTA *via* RAFT solution polymerization

GMA (16.8 g, 105 mmol), CPDB (0.415 g, 1.50 mmol), and AIBN (49.0 mg, 0.30 mmol; CPDB/AIBN molar ratio = 5.0) were weighed into a 100 mL round-bottomed flask. Anhydrous ethanol (21.0 g, previously purged with nitrogen for 1 h) was then added to produce a 45% w/w solution, which was placed in an ice bath and purged under nitrogen for 30 min. The sealed flask was immersed in an oil bath set at 70 °C to initiate the RAFT solution polymerization of GMA and stirred for 2 h at this temperature. The GMA polymerization was then quenched by exposure to air, followed by cooling the reaction mixture to room temperature. Ethanol (25 mL) was added to dilute the reaction solution, followed by precipitation into a ten-fold excess of dichloromethane in order to remove unreacted GMA monomer. The precipitate was isolated *via* filtration and washed with excess dichloromethane before being dissolved in methanol (50 mL). The crude polymer was precipitated for a second time by addition to excess dichloromethane and isolated by filtration. It was then dissolved in water and freeze-dried for 48 h to afford a pink powder.

#### Synthesis of PGMA_45_–PHPMA_*x*_ diblock copolymer worms or vesicles *via* RAFT aqueous dispersion polymerization of HPMA

A typical protocol for the synthesis of PGMA_45_–PHPMA_115_*via* RAFT aqueous dispersion polymerization of HPMA using the PGMA_45_ macro-CTA is as follows: PGMA_45_ macro-CTA (0.15 g, 0.02 mmol), HPMA monomer (0.35 g, 2.3 mmol), and ACVA (1.5 mg, 5.0 μmol; PGMA_45_ macro-CTA/ACVA molar ratio = 4.0) were added to a 25 mL round-bottomed flask, prior to addition of water to produce a 15% w/w solution. This reaction solution was purged with nitrogen gas for 30 min at 20 °C prior to immersion into an oil bath set at 70 °C. The reaction mixture was stirred for 3 h to ensure essentially complete conversion of the HPMA monomer, then the polymerization was quenched by exposure to air, followed by cooling to ambient temperature. For the synthesis of PGMA_45_–PHPMA_165_ vesicles, the mass of added HPMA monomer was increased to 0.50 g, and the volume of water was adjusted accordingly to maintain a constant 15% w/w solids.

#### Morphological transitions for PGMA_45_–PHPMA_*x*_ diblock copolymer nano-objects

The initial 15% w/w aqueous copolymer vesicle (or worm) dispersion was diluted to 3.0% w/w using water and adjusted to pH 10 by addition of 0.02 M NaOH solution. CPBA was dissolved in either 1 M or 0.1 M NaOH solution to produce a 6.0%, 3.0%, 1.0% or 0.5% w/w solution at pH 10. These four alkaline CPBA solutions were stored in the dark prior to use. 1.0 g of the 3.0% w/w aqueous vesicle (or worm) dispersion was then mixed with each CPBA solution in turn at the desired volumetric ratio in a 10 mL vial, and the resulting mixture was further diluted using either aqueous NaOH or water to produce a 1.0% (or 0.77%) w/w aqueous dispersion of copolymer nano-objects (0.23% w/w with respect to the PGMA stabilizer block) at pH 10. The sealed vial was stored at room temperature and aged for the desired time period prior to turbidimetry, TEM, zeta potential and DLS studies.

### Characterization techniques

#### NMR spectroscopy

^1^H NMR spectra were recorded in CD_3_OD using a 400 MHz Bruker Avance-500 spectrometer (64 scans averaged per spectrum). ^11^B NMR spectra were recorded in deionized water at the desired pH using quartz NMR tubes on a 500 MHz Bruker Avance III HD spectrometer operating at 160.46 MHz (typically 88 scans were averaged per spectrum).

#### Gel permeation chromatography (GPC)

Polymer molecular weights and dispersities were determined using a DMF GPC set-up operating at 60 °C and comprising two Polymer Laboratories PL gel 5 μm Mixed-C columns connected in series to a Varian 390-LC multidetector suite (only the refractive index detector was utilized) and a Varian 290-LC pump injection module. The GPC eluent was HPLC-grade DMF containing 10 mM LiBr at a flow rate of 1.0 mL min^–1^. Calibration was conducted using a series of ten near-monodisperse poly(methyl methacrylate) standards (*M*_n_ = 625–2 480 000 g mol^–1^). Aqueous copolymer dispersions were freeze-dried overnight to obtain powders. Copolymer solutions (0.70% w/w) were prepared in DMF containing DMSO (1.0% v/v) as a flow rate marker. Chromatograms were analyzed using Varian Cirrus GPC software (version 3.3).

#### Dynamic light scattering (DLS)

DLS studies were conducted on 1.0% (or 0.77%) w/w copolymer dispersions at 20 °C using a Malvern Instruments Zetasizer Nano series instrument equipped with a 4 mW He–Ne laser (*λ* = 633 nm) and an avalanche photodiode detector. Scattered light was detected at 173°. For aqueous electrophoresis measurements, copolymer dispersions were diluted to 0.2% w/w using dilute aqueous NaOH containing the same CPBA concentration and also 1 mM KCl as background electrolyte prior to immediate analysis. Intensity-average hydrodynamic diameters were calculated *via* the Stokes–Einstein equation, while zeta potentials were determined *via* the Henry equation using the Smoluchowski approximation.

#### Transmission electron microscopy (TEM)

Copper TEM grids (Agar Scientific, UK) were surface-coated in-house to yield a thin film of amorphous carbon. The grids were then plasma glow-discharged for 30 s to create a hydrophilic surface. Aqueous dispersions of copolymer nano-objects were diluted to 0.2% w/w using the same solvent and a 5 μL droplet of the diluted dispersion was placed on a grid immediately for 10 s and then blotted with filter paper to remove excess solution. To stain the aggregates, a 5 μL droplet of 0.75% w/w uranyl formate solution was soaked on the sample-loaded grid for 40 s and then carefully blotted to remove excess stain. The grids were then dried using a vacuum hose. Imaging was performed at 80 kV using a FEI Tecnai Spirit microscope equipped with a Gatan 1kMS600CW CCD camera.

#### Turbidimetry studies

Transmittance measurements of 1.0% w/w aqueous copolymer dispersions were recorded at 20 min intervals over 24 h using a Shimadzu UV-1800 spectrometer operating at 20 °C using a fixed wavelength of 450 nm.

## Results and discussion

A PGMA_45_ (the subscript refers to its mean degree of polymerization, DP) macromolecular chain transfer agent was synthesized by reversible addition–fragmentation chain transfer (RAFT) solution polymerization^52^ and subsequently used for the RAFT aqueous dispersion polymerization of 2-hydroxypropyl methacrylate (HPMA) to produce well-defined PGMA_45_–PHPMA_*x*_ nano-objects. Unlike traditional self-assembly approaches based on post-polymerization processing in dilute solution, such PISA formulations enable the convenient preparation of well-defined diblock copolymer nano-objects at relatively high solids, *e.g.* 15% w/w in this study. The morphology of block copolymer nano-objects primarily depends on the fractional packing parameter (*P*), which can be tuned by systematically varying the DP of the core-forming PHPMA block in PISA syntheses.^56^ Mean DPs (or *x* values) of 115 and 165 were targeted for the PHPMA block so as to afford worms or vesicles, respectively. Gel permeation chromatography (GPC; DMF eluent) analyses indicated relatively high blocking efficiencies and low final dispersities (*M*_w_/*M*_n_ ≤ 1.16) for these PGMA–PHPMA diblock copolymers (see Fig. S1a in ESI†). As expected, a free-standing soft hydrogel was obtained for PGMA_45_–PHPMA_115_ while PGMA_45_–PHPMA_165_ formed a free-flowing turbid dispersion. Transmission electron microscopy (TEM) studies confirmed a well-defined worm or vesicle morphology, respectively (see Fig. S1b†).

**Fig. 1:**
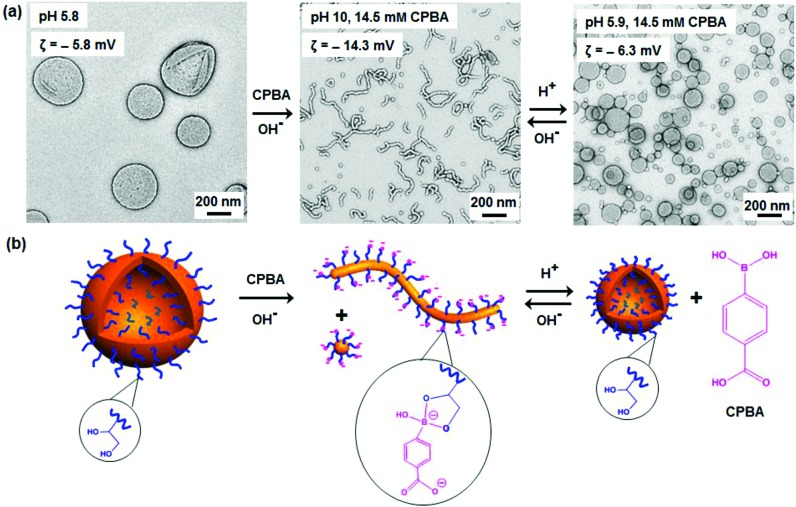
(a) TEM images obtained for PGMA_45_–PHPMA_165_ nano-objects before and after morphological transitions performed under various conditions. (b) Schematic cartoon depicting the dynamic covalent chemistry that drives such morphological transitions.

**Fig. 2:**
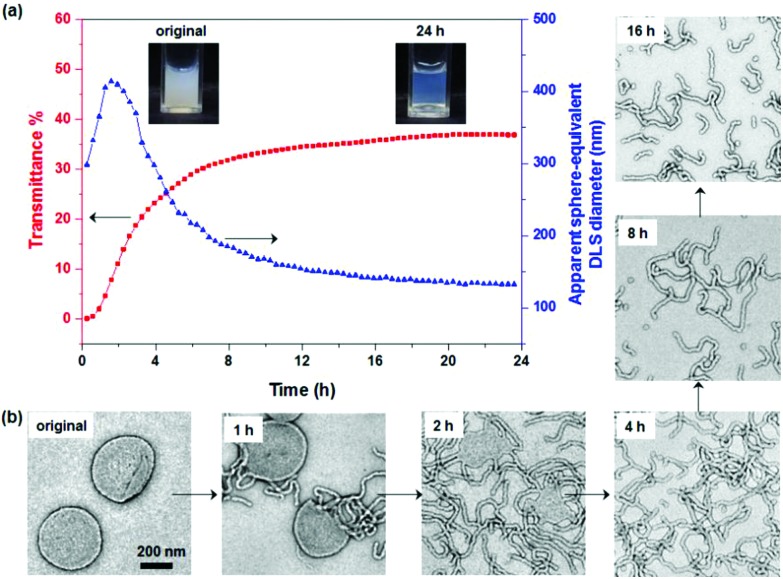
(a) Transmittance (red curve) and apparent sphere-equivalent DLS diameter (blue curve) obtained for the series of 1.0% w/w PGMA_45_–PHPMA_165_ nano-objects generated on ageing for 24 h after addition of 14.5 mM CPBA at pH 10. Insets show two digital photographs recorded for the original dispersion and also the final dispersion after ageing for 24 h in the presence of CPBA at pH 10. (b) TEM images recorded at various time points illustrating the evolution from the initial vesicles to worms/spheres. The 200 nm scale bar shown for the TEM image obtained for the original vesicles applies to all the other images shown.

Morphological transitions of a 1.0% w/w aqueous dispersion of PGMA_45_–PHPMA_165_ vesicles were studied in the presence of 14.5 mM CPBA (CPBA/GMA molar ratio *r* = 1.0) at approximately pH 10 (complexation of CPBA with 1,2-diols can cause a slight reduction in the initial solution pH^57^). The original vesicles were transformed into worms (plus a minor fraction of spheres) after being aged at 20 °C for 24 h in the presence of CPBA (see Fig. 1a). In contrast, no change in the original vesicular morphology occurred under the same conditions in the absence of CPBA (see Fig. S2a†). The CPBA-induced morphological transition was confirmed by dynamic light scattering (DLS) studies (see Fig. S2b†). TEM, DLS and turbidimetry studies provided useful further insights regarding the evolution in morphology (see Fig. 2). The vesicle-to-worm transition is known to proceed *via* jellyfish, octopi and branched worms and these transient intermediates can be observed by TEM. DLS experiments indicated an initial modest increase in size (corresponding to the formation of jellyfish), followed by a significant reduction as relatively short worms are eventually generated, while a gradual increase in transmittance was observed by turbidimetry. The vesicle-to-worm transition can be attributed to the formation of phenylboronate ester bonds between the CPBA and the PGMA stabilizer chains.^52^ Such dynamic covalent chemistry reduces the fractional packing parameter because: (i) the overall mass of the stabilizer chains increases and (ii) the formation of each phenylboronate ester introduces two anionic charges, so the stabilizer chain becomes a pseudo-polyelectrolyte and hence expands to occupy a larger volume. Aqueous electrophoresis measurements provided supporting evidence for the expected change in nanoparticle surface charge: the original vesicles had a zeta potential of –5.8 mV at pH 5.8, which increased to –14.3 mV in the presence of CPBA at pH 10. In contrast, only a marginal increase in negative zeta potential to –8.5 mV was observed in the absence of CPBA at pH 10.

**Scheme 2:**
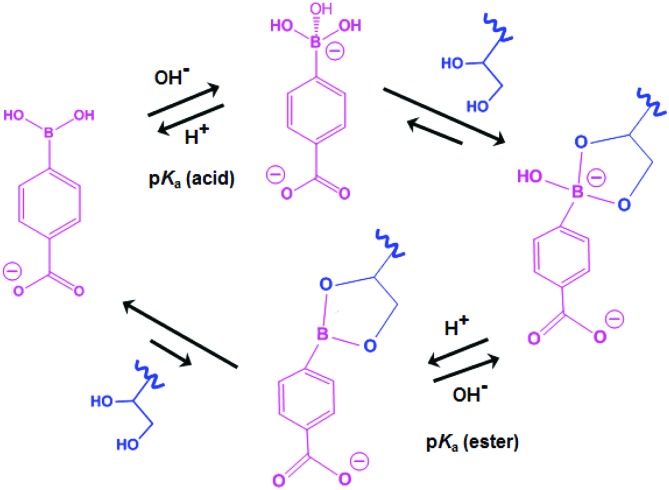
Complex equilibria between CPBA and the 1,2-diol units on the PGMA stabilizer chains in aqueous solution.

**Fig. 3:**
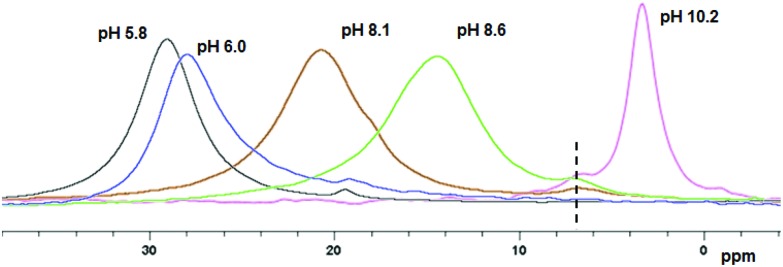
^11^B NMR spectra obtained for 62.5 mM CPBA in the presence of 1.0% PGMA_45_ macro-CTA (*r* = 1.0), which were recorded after 24 h at various pH (see labels). (pH labelled are real-time values after 24 h).

In a second control experiment, no morphological transition was observed for PGMA_45_–PHPMA_165_ vesicles (see Fig. S3†) when CPBA was added at pH 5.8 (the original pH of the vesicle dispersion). Essentially no phenylboronate ester bonds are formed at this relatively low pH. This is because the phenylboronic acid species must be deprotonated to form a phenylboronate anion prior to its complexation with 1,2-diol units.^58^ The pH-dependent equilibria between CPBA and PGMA in aqueous solution is shown in Scheme 2. The p*K*_a_ of CPBA is 8.35,^59^ so only 10% phenylboronate anion is formed at pH 7.4.^43^ A higher solution pH converts phenylboronic acid into phenylboronate anion, thus enabling CPBA binding to the PGMA stabilizer chains, as confirmed by the prominent phenylboronate ester signal observed at 6.8 ppm in the ^11^B NMR spectrum (see Fig. 3). This explains the morphological transitions in alkaline solution observed by TEM (see Fig. S3†). It is emphasized that such dynamic covalent chemistry is fully reversible. As shown in Scheme 2, when the solution pH is below its p*K*_a_, the phenylboronate ester is converted into an unstable intermediate species (phenylboronic ester), which then forms CPBA.^45^ This is confirmed by the disappearance of the ^11^B NMR signal at 6.8 ppm on lowering the solution pH from 10.2 to 6.0 (see pink and blue curves in Fig. 3). This reversible binding on switching pH should drive a reversible morphological transition for the nano-objects. Indeed, TEM studies indicate that the worms are transformed into vesicles on switching pH from pH 10 to approximately pH 6 (see Fig. 1a and S4a†), while the zeta potential reverts to –6.3 mV. Once CPBA is no longer bound to the PGMA stabilizer chains, it can be removed *via* dialysis, as confirmed by ^11^B NMR spectroscopy (see red curve in Fig. S4a†). As expected, the vesicular morphology remained unchanged after dialysis against water at pH 6.5, see Fig. S4a.†

The reconstituted vesicles are significantly smaller and less polydisperse than the original vesicles (see DLS data shown in Fig. S4b†). Similar observations were reported by Warren *et al.* for thermoresponsive poly(ethylene glycol)–PHPMA vesicles.^60^ A tentative explanation is that the stochastic aggregation of the worms leads to lower vesicle aggregation numbers in dilute solution – in contrast, the original relatively large vesicles were prepared *via* PISA at 15% w/w copolymer concentration. Indeed, at 2.0% w/w concentration, this reversible transition generated somewhat larger vesicles (see Fig. S5a†). However, if the same pH cycle is performed for a 5.0% w/w dispersion, a heterogeneous insoluble paste was obtained after returning the solution to pH 6 (see inset photo, Fig. S5b†). TEM analysis indicated the presence of small vesicles within the aqueous supernatant (see Fig. S5b†). When the same morphological transition was conducted at 10% w/w concentration, the initial free-flowing turbid vesicular dispersion was first converted into a free-standing hydrogel, which then formed a less turbid, free-flowing viscous dispersion (see Fig. S5c†). These observations suggest a sequential two-step vesicle-to-worm-to-sphere transition. On switching the solution pH from 10 to 6, the viscous dispersion initially became a free-standing gel which then rapidly formed an insoluble paste, rather than a free-flowing turbid vesicular dispersion. This indicates that the spheres can be converted into worms, but the latter cannot form vesicles at high copolymer concentration. Indeed, there appears to be a significant kinetic barrier for the worm-to-vesicle transition under such conditions.^20^

**Fig. 4:**
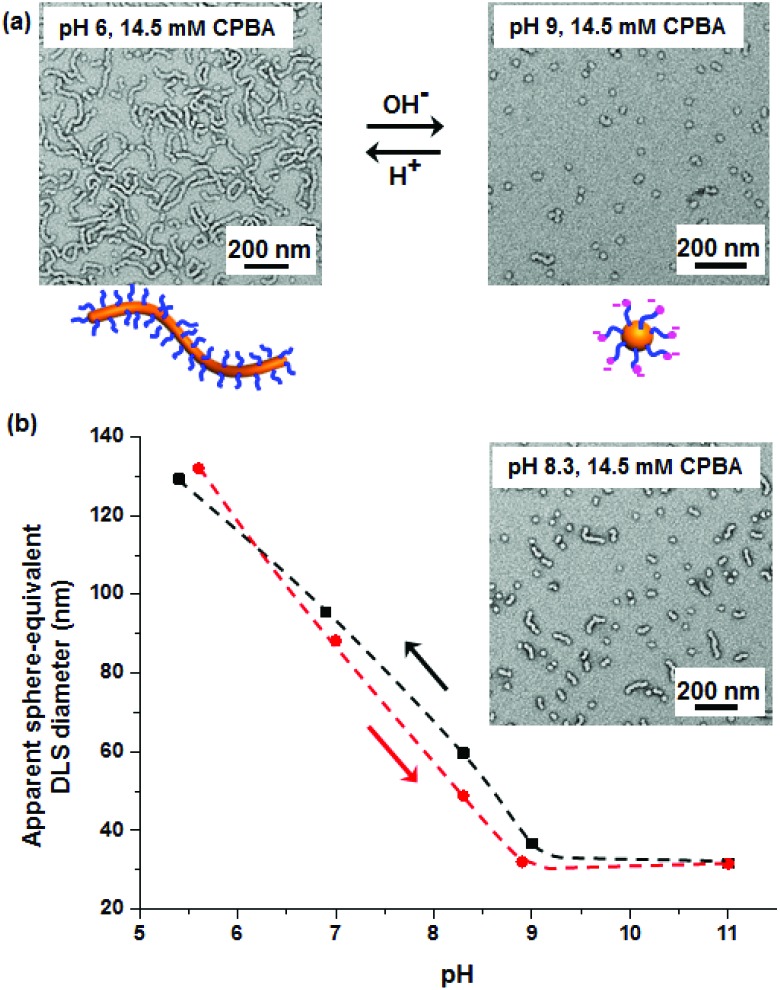
(a) TEM images recorded after 24 h for the reversible worm-to-sphere transition exhibited by a 0.77% w/w aqueous dispersion of PGMA_45_–PHPMA_115_ nano-objects on changing the solution pH in the presence of 14.5 mM CPBA (*r* = 1.0), with two corresponding schematic cartoons. (b) Change in sphere-equivalent DLS diameter on adjusting the solution pH, illustrating good reversibility for the worm-to-sphere transition (inset: TEM image recorded for the copolymer dispersion obtained at pH 8.3).

The reversible worm-to-sphere transition was also investigated for a 0.77% w/w PGMA_45_–PHPMA_115_ dispersion in the presence of 14.5 mM CPBA at pH 9 (see Fig. 4 and S6†). Bearing in mind that DLS reports a sphere-equivalent diameter for worms, this technique can be used to monitor the pH-induced worm-to-sphere transition *in situ* by determining the apparent nano-object dimensions (see Fig. 4b). The sphere-equivalent DLS diameter decreases monotonically on adjusting the solution pH from 5.6 to 8.9. A mixture of spheres and short worms are present at pH 8.3 (see inset in Fig. 4b). As expected, almost no further change in size occurred between pH 8.9 and pH 11. Gradually lowering the pH *via* HCl addition indicates excellent reversibility for the worm-to-sphere-to-worm transitions (see Fig. 4a and b). In contrast, control experiments confirm that no such morphological transitions are observed in the absence of CPBA (see Fig. S6b†).

**Fig. 5:**
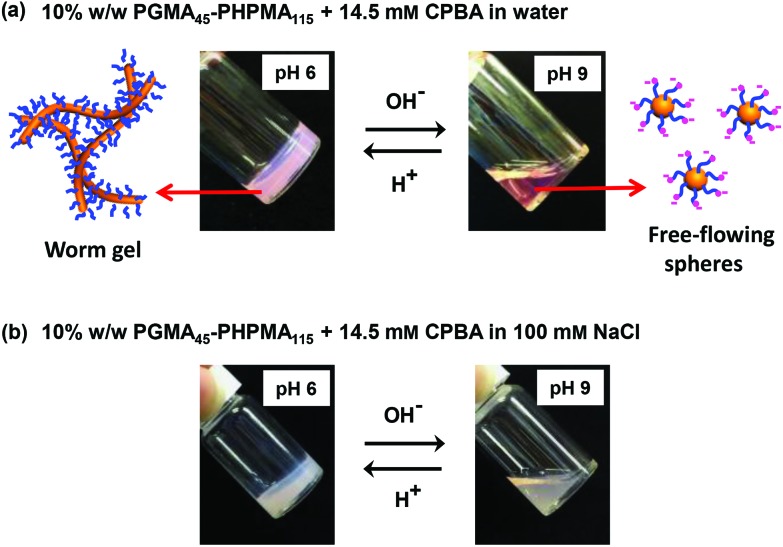
(a) and (b) Digital photographs illustrating the reversible sol–gel transitions obtained for a 10% w/w aqueous dispersion of PGMA_45_–PHPMA_115_ nano-objects in the presence of 14.5 mM CPBA and either the absence or presence of 100 mM NaCl.

Unlike the vesicle-to-worm transition, the worm-to-sphere transition is reversible at high copolymer concentration, which enables (de)gelation to occur under these conditions. At 10% w/w copolymer, the worms form a soft hydrogel at around pH 6 as a result of multiple inter-worm interactions, while the corresponding non-interacting spheres form a free-flowing aqueous dispersion at approximately pH 9. Therefore, the CPBA-induced reversible worm-to-sphere transition leads to (de)gelation under these conditions on switching pH (see Fig. 5a). Interestingly, this pH-modulated transition differs from pH-responsive transitions previously reported by Armes and co-workers, where worm-to-sphere transitions were completely suppressed in the presence of 100 mM salt.^20^ In contrast, CPBA-induced worm-to-sphere transitions enable reversible (de)gelation to occur in the presence of 100 mM NaCl (see Fig. 5b). In principle, such salt-tolerance should extend the scope of potential applications. Moreover, the mildly alkaline pH required for phenylboronic acid binding to the PGMA stabilizer chains can be lowered to around neutral pH by selecting alternative phenylboronic acid derivatives with appropriate substituents.^61^ Such refinements are likely to be required for biomedical applications.

## Conclusions

In summary, dynamic covalent chemistry is exploited to trigger reversible morphological transitions in aqueous dispersions of diblock copolymer nano-objects. These transitions are achieved by the reversible formation of a phenylboronate ester bond between CPBA and the pendent 1,2-diol groups on the PGMA stabilizer chains by varying the solution pH. The vesicle-to-worm transition is reversible at a relatively low copolymer concentration, but becomes irreversible at higher concentrations. In contrast, the worm-to-sphere transition is reversible over a wide range of copolymer concentration, which leads to *in situ* (de)gelation at higher concentrations. The dynamic covalent chemistry strategy described herein offers considerable scope for designing new stimulus-responsive block copolymer nano-objects and associated hydrogels that can respond to changes in their local environment in the presence of salt.

## Supplementary Material

Supplementary information
