# Supplementary material for: Stimulus-responsive block copolymer nano-objects and hydrogels via dynamic covalent chemistry
Source: Polym Chem. 2017 Jul 28;8(35):5374–80. doi: 10.1039/c7py01242j (PMC5735357; doi:10.1039/c7py01242j)
Supplement: Supplementary file 1 [file PY-008-C7PY01242J-s001.pdf]

Electronic Supplementary Material (ESI) for Polymer Chemistry.
This journal is © The Royal Society of Chemistry 2017

Supporting Information for:

Stimulus-responsive Block Copolymer Nano-Objects and Hydrogels via Dynamic Covalent Chemistry

Renhua Deng,* Yin Ning, Elizabeth R. Jones, Victoria J. Cunningham, Nicholas J. W. Penfold, and Steven P. Armes*

Supporting Figures

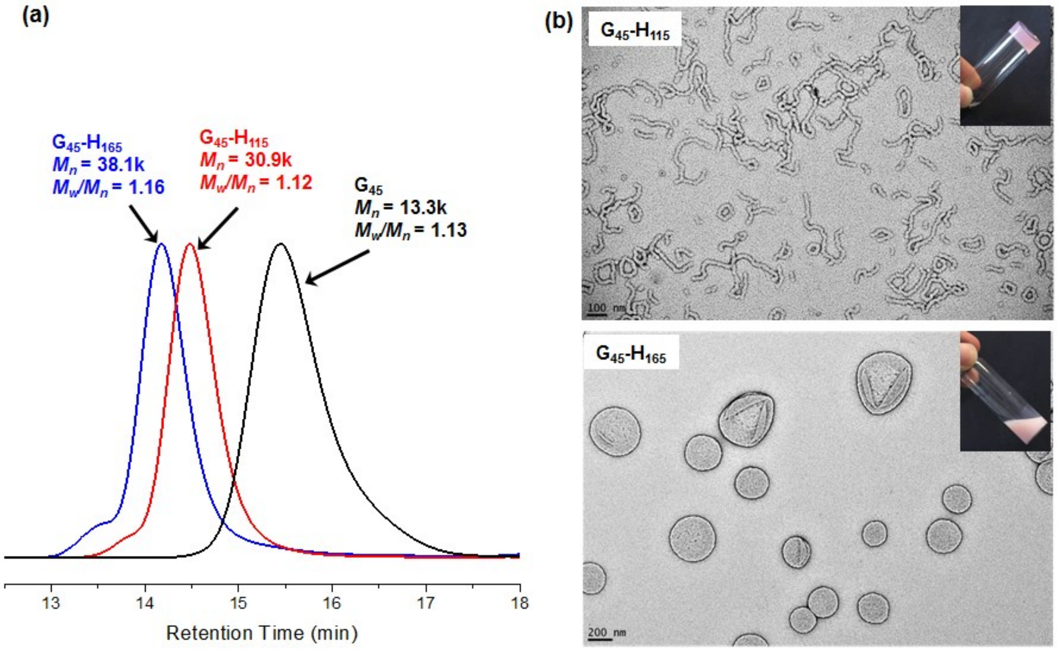

Figure S1. (a) DMF GPC curves obtained for the $PGMA_{45}$ macro-CTA precursor (black curve) and the corresponding $PGMA_{45}$-$PHPMA_{115}$ and $PGMA_{45}$-$PHPMA_{165}$ diblock copolymers. (b) Representative TEM images obtained for $PGMA_{45}$-$PHPMA_{115}$ worms and $PGMA_{45}$-$PHPMA_{165}$ vesicles, respectively. Insets are digital photographs of the visual appearance of these corresponding aqueous dispersions recorded at 15% w/w solids (the pink color originates from the CPDB RAFT agent). Under these conditions, the worms form a soft free-standing gel, whereas the vesicles form a free-flowing fluid.

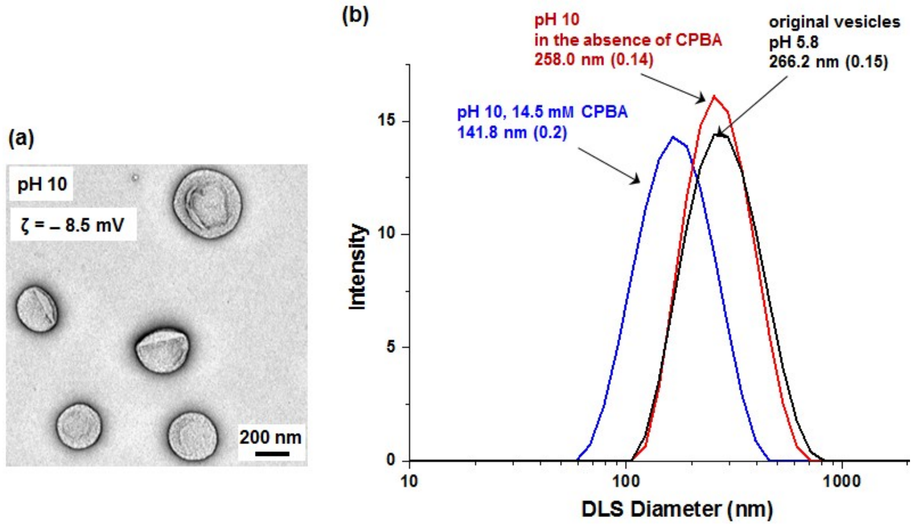

Figure S2. (a) TEM image obtained for a 1.0% w/w aqueous dispersion of $PGMA_{45}$-$PHPMA_{165}$ nano-objects after ageing for 24 h in the absence of CPBA at pH 10. (b) DLS particle size distribution obtained for a 1.0% w/w aqueous dispersion of as-synthesized $PGMA_{45}$-$PHPMA_{165}$ vesicles at pH 5.8 (black curve), and after ageing for 24 h at pH 10 in the absence (red curve) and presence (blue curve) of 14.5 mM CPBA.

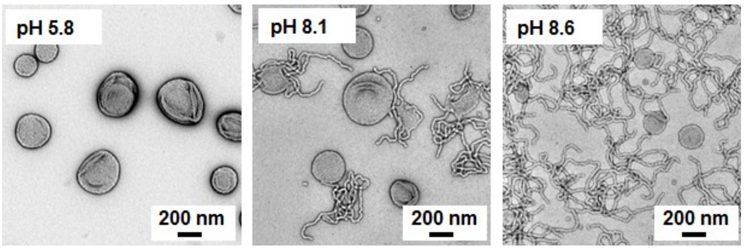

Figure S3. TEM images recorded for 1.0% w/w $PGMA_{45}$-$PHPMA_{165}$ nano-objects at various solution pH in the presence of 14.5 mM CPBA (r = 1.0). Labels indicate pH values recorded after ageing for 24 h.

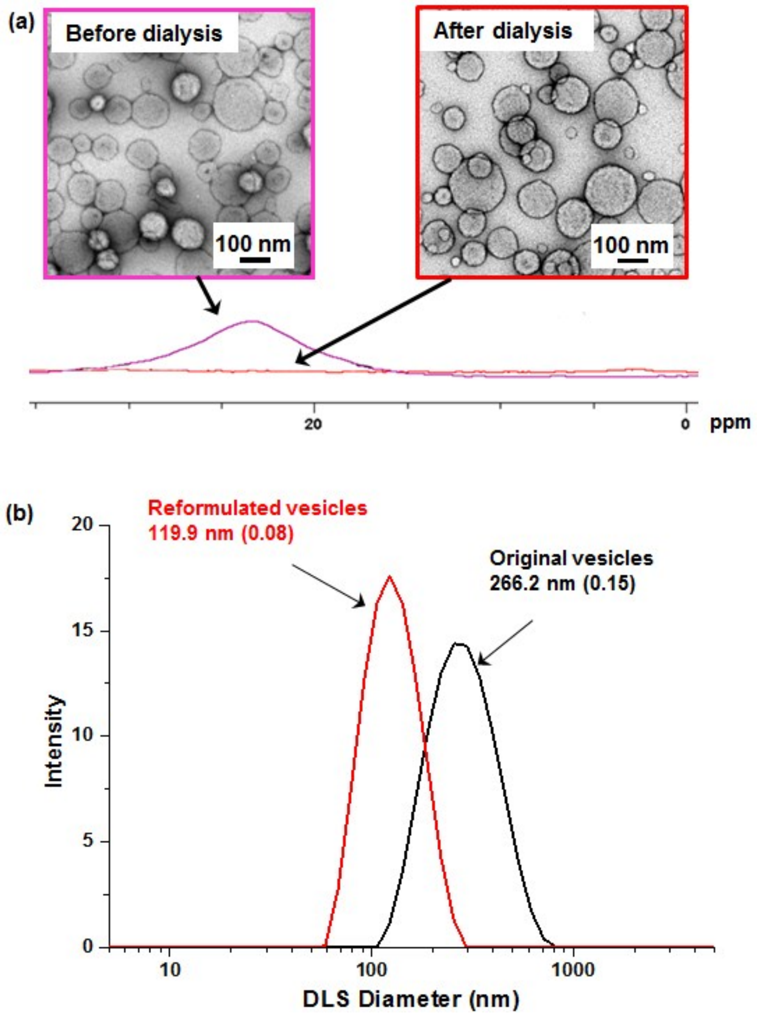

Figure S4. (a) TEM images and corresponding ^{11}B NMR spectra obtained for a 1.0% w/w aqueous dispersion of $PGMA_{45}$-$PHPMA_{165}$ diblock copolymer nano-objects after a pH switch from 10 to 6.5: in the presence of 14.5 mM CPBA before dialysis (pink) and in the absence of CPBA after dialysis (red). (b) DLS particle size distributions obtained for a 1.0% w/w aqueous dispersion of $PGMA_{45}$-$PHPMA_{165}$ vesicles, where the black curve represents originally prepared vesicles at pH 5.8, and the red one represents reformulated vesicles via adjusted pH from 5.8 to 10 (transformed into worms) and then returned to pH 6.0 in the presence of 14.5 mM CPBA.

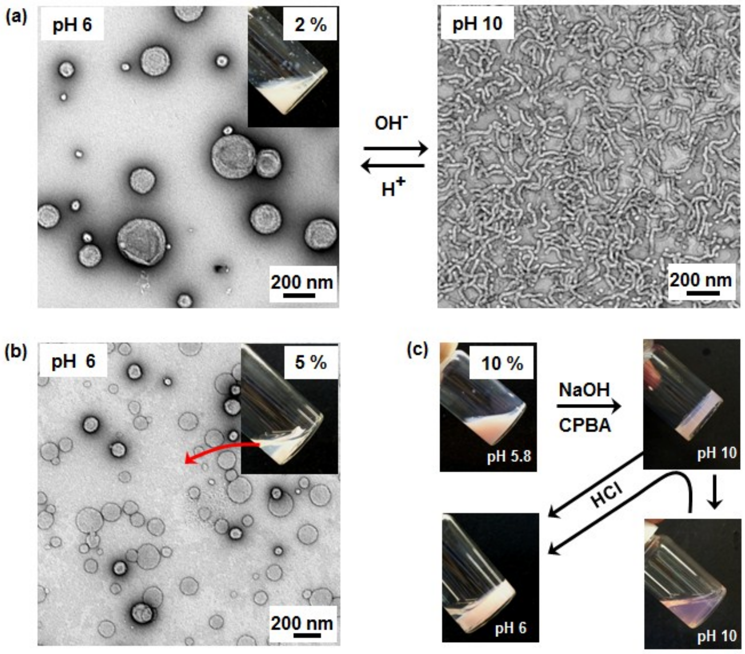

Figure S5. (a) TEM images recorded for a 2.0% w/w aqueous dispersion of $PGMA_{45}$-$PHPMA_{165}$ nano-objects on switching from pH 6 to pH 10 in the presence of 14.5 mM CPBA after ageing for 24 h. The initial vesicles observed at pH 6 are converted into worms on adjusting the solution pH to pH 10. Addition of acid confirms the reversibility of this transition. (b) TEM image obtained for the aqueous supernatant comprising a 5.0% w/w aqueous dispersion of $PGMA_{45}$-$PHPMA_{165}$ diblock copolymer nano-objects after the solution pH was adjusted from 10 to 6 followed by ageing for 24 h in the presence of 14.5 mM CPBA. (c) Digital photographs illustrating the *irreversible* vesicle-to-worm (or vesicle-to-sphere) transition exhibited by 10% w/w $PGMA_{45}$-$PHPMA_{165}$ nano-objects in the presence of 14.5 mM CPBA.

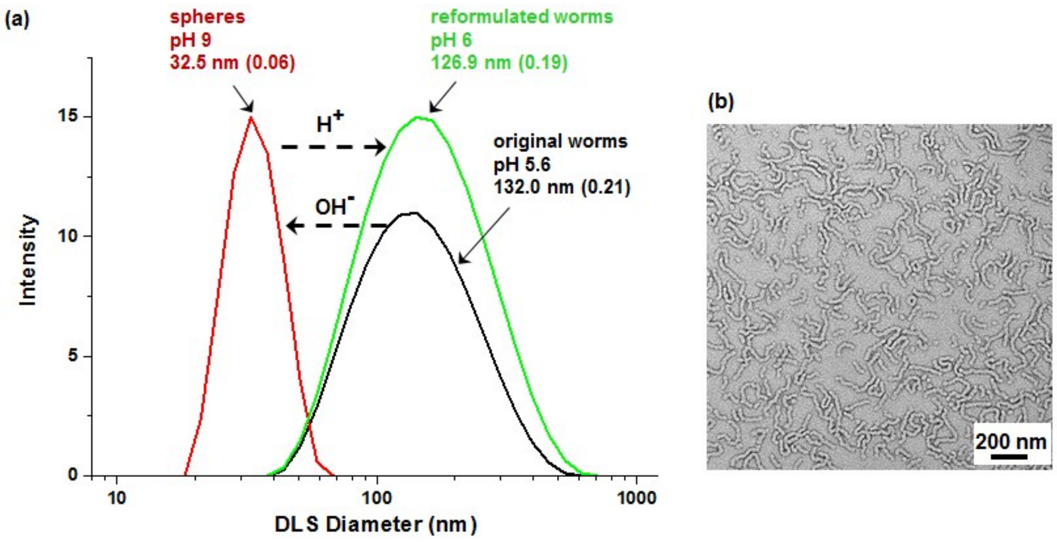

Figure S6. (a) DLS particle size distributions recorded for a 0.77% w/w aqueous dispersion of $PGMA_{45}$-$PHPMA_{115}$ nano-objects on switching pH in the presence of 14.5 mM CPBA (r = 1.0) both initially at pH 5.6 and after ageing for 24 h at pH 8.9, followed by a switch back to pH 6. These DLS data are consistent with a reversible worm-to-sphere transition occurring under alkaline conditions. (b) TEM image obtained for the same 0.77% w/w aqueous dispersion of $PGMA_{45}$-$PHPMA_{115}$ nano-objects at pH 9 in the absence of CPBA after ageing for 24 h.
